# Fine Tuning of Traumatic Brain Injury Management in Neurointensive Care—Indicative Observations and Future Perspectives

**DOI:** 10.3389/fneur.2021.638132

**Published:** 2021-02-24

**Authors:** Teodor M. Svedung Wettervik, Anders Lewén, Per Enblad

**Affiliations:** Department of Neuroscience, Section of Neurosurgery, Uppsala University, Uppsala, Sweden

**Keywords:** multimodality monitoring, secondary brain injury, secondary insults, neurointensive care, traumatic brain injury

## Abstract

Neurointensive care (NIC) has contributed to great improvements in clinical outcomes for patients with severe traumatic brain injury (TBI) by preventing, detecting, and treating secondary insults and thereby reducing secondary brain injury. Traditional NIC management has mainly focused on generally applicable escalated treatment protocols to avoid high intracranial pressure (ICP) and to keep the cerebral perfusion pressure (CPP) at sufficiently high levels. However, TBI is a very heterogeneous disease regarding the type of injury, age, comorbidity, secondary injury mechanisms, etc. In recent years, the introduction of multimodality monitoring, including, e.g., pressure autoregulation, brain tissue oxygenation, and cerebral energy metabolism, in addition to ICP and CPP, has increased the understanding of the complex pathophysiology and the physiological effects of treatments in this condition. In this article, we will present some potential future approaches for more individualized patient management and fine-tuning of NIC, taking advantage of multimodal monitoring to further improve outcome after severe TBI.

## Introduction

In the later decades of the twentieth century, the understanding of the critical factors responsible for neurological deterioration after traumatic brain injury (TBI) increased (1). It became clear that although the primary brain injury after TBI cannot be cured, secondary insults leading to further brain injury can be avoided by vigilant monitoring with attentive prevention and early treatment of such insults. This led to the development of standardized management protocols for severe TBI at specialized neurointensive care (NIC) units, resulting in significant improvements in the clinical outcome for these patients (2, 3).

**Figure 1:**
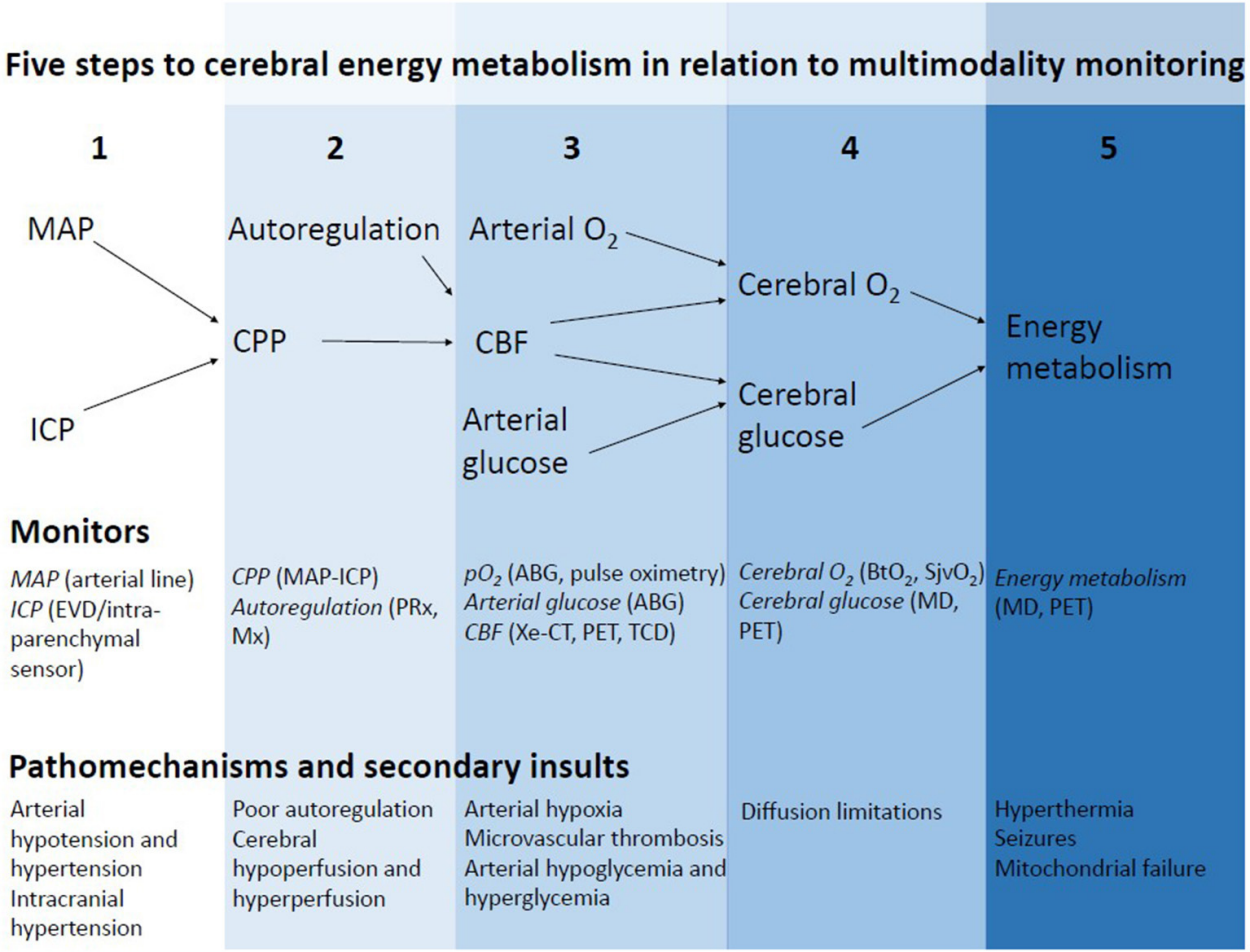
Five critical steps in the pathway to cerebral energy metabolism in relation to multimodality monitoring. By monitoring all the steps in this pathway to achieve sufficient cerebral energy metabolism, energy failure may be detected in time and the correct diagnosis can be made. Treatments should aim at counteracting the specific mechanism that causes cerebral energy metabolic disturbances.

The concept of NIC, to avoid secondary brain injury by intensive monitoring and aggressive treatment of emerging secondary insults, is still the fundamental basis for modern NIC. The purpose of this presentation was to give an overview of potential directions toward the NIC of the future. The structure of this review is based on the critical steps to achieve sufficient cerebral energy metabolism for neuronal survival in relation to multimodality monitoring (Figure 1).

## Traditional Treatment Targets in Neurointensive Care

### Intracranial Pressure—Step 1

In the early NIC, the main focus was to avoid high intracranial pressure (ICP). The basic understanding of ICP dynamics was derived from the Monro–Kellie hypothesis, indicating that ICP will increase due to the progression of posttraumatic intracranial hemorrhages or brain edema when there is no more compensatory reserve to reduce any other intracranial volume (4). Severe intracranial hypertension leads to brain herniation syndromes, cerebral hypoperfusion, and, ultimately, fatal outcome if untreated. In awake TBI patients, progressing intracranial hypertension may be detected by clinical evaluation as neurological deterioration, but it may be much more difficult to detect this in time in already comatose patients who are intubated and sedated.

Invasive ICP monitoring was introduced in the 1950s by Guillaume (5), further developed in the 1960s by Lundberg (6), and has ever since been used in NIC (Tables 1, 2, Figure 1). Over the years, the definition of what ICP threshold dichotomizes acceptable and dangerous ICP elevation has been extensively debated (7, 13, 14), and the value of ICP monitoring has also been questioned (15). However, the current Brain Trauma Foundation (BTF) guidelines recommend ICP monitoring in unconscious TBI patients and suggest an upper threshold at 22 mmHg (7). In the case of higher ICP, lowering therapies such as evacuation of significant intracranial hematomas, mild hyperventilation, cerebrospinal fluid (CSF) drainage, barbiturates, and decompressive craniectomy may be used in tiered protocols (7, 16, 17).

**Table 1:** Multimodality monitoring—a selection of methods and their benefits and limitations.

**Physiological variable**	**Monitoring method**	**Continuous/Intermittent**	**Global/Focal**	**Benefits**	**Limitations**
ICP	EVD	Continuous	Global	Both monitoring and treatment of ICP by CSF drainage.	Infection risk. Impossible when compressed ventricles. Risk of invalid ICP monitoring in case of slit ventricle.
	Intraparenchymal sensor	Continuous	Global	Possible also when compressed ventricles. Invalid ICP monitoring due to slit ventricle is not a problem.	Does not offer ICP treatment with CSF drainage.
CPP	ICP device and arterial line for continuous systemic ABP.	Continuous	Global	Feasible, continuous global CBF surrogate.	Unreliable surrogate, does not consider cerebrovascular resistance.
CBF autoregulation	PRx (ICP and ABP)	Continuous with time window	Global	Feasible measure of the global autoregulatory status.	Low signal-to-noise ratio. Does not take into account focal asymmetries.
	CPPopt (PRx and CPP)	Continuous with time window	Global	Feasible, continuous global CBF surrogate.	Frequent absence of U-shaped curves. Does not take into account focal asymmetries. Requires advanced software.
	Mx (TCD and CPP)	Intermittent	Global and focal	May detect regional autoregulatory differences.	User-dependent. Poor evaluation of the posterior circulation.
Cerebral blood flow	Radiology (Xe-CT, PET, MRI)	Intermittent	Global and focal	Both global and focal.	Difficult to transport unstable patients to the radiology department.
	TCD	Intermittent	Global and focal	Feasible and may be used bedside.	Measures velocity. User-dependent. Poor evaluation of the posterior circulation.
	Intraparenchymal thermal diffusion probe	Continuous	Focal	Feasible and continuous CBF measure.	Unreliable. Focal, does not take into account variabilty in CBF between brain regions.
Brain tissue oxygenation	Jugular bulb catheter (SjvO_2_)	Continuous	Global	Continuous and feasible global measure.	Low sensitivity for focal ischemia.
	Intraparenchymal device (BtO_2_)	Continuous	Focal	Continuous and feasible focal measure.	Variabilty in oxygenation between brain regions.
	NIRS	Continuous	Focal	Non-invasive.	Unreliable. Does not evaluate the posterior circulation.
Cerebral energy metabolism	MD	Continuous	Focal	Feasible for continuous evaluation.	Variability in energy metabolism between brain regions.
	PET	Intermittent	Global and focal	Possible to investigate complex aspects of energy metabolism.	Difficult to transport unstable patients to the radiology department.

*ABP, Arterial blood pressure; CBF, Cerebral blood flow; CSF, Cerebrospinal fluid; EVD, external ventricular drain; ICP, Intracranial pressure; MD, Microdialysis; Mx, Mean flow inex; NIRS, Near infrared spectroscopy; PET, Positron emission tomography; PRx, Pressure reactivity index; TCD, Transcranial Doppler; Xe-CT, Xenon-enhanced computed tomography*.

**Table 2:** Monitoring variables, target intervals, and treatments.

**Variable**	**Target interval**	**Treatment**
ICP	BTF: ICP ≤ 22 mm Hg (7) Uppsala: ICP ≤ 20 mm Hg (2)	Head elevation Hematoma evacuation Hyperventilation CSF drainage Sedation Barbiturates Decompressive craniectomy
CPP	BTF: 60 to 70 mm Hg (7) Uppsala: CPP ≥ 60 mm Hg (2) Lund concept: CPP 50 to 70 mm Hg (8) Autoregulatory management: CPP close to CPPopt (9)	ICP control (above) Intravenous fluids Vasopressors
CBF pressure autoregulation	BTF: No target (7) Uppsala: No target (2)	CPP optimum Hyperventilation Hyperoxia Body temperature control Arterial glucose and lactate management
CBF	BTF: No target (7) Uppsala: No target (2)	CPP and autoregulation management
Arterial oxygenation	BTF: No target (7) Uppsala: pO_2_ > 12 kPa and Hgb > 100 g/L (2)	Respiratory optimization Red blood cell traunsfusion
Arterial glucose	BTF: No target (7) Uppsala: 5–10 mM (2) Tight glycemic control: 4.4–6.1 mM (10)	Intravenous glucose Insulin injection/infusion
Cerebral oxygenation	BTF: No target (7) Uppsala: No target (2)	CBF and arterial oxygenation management
Cerebral glucose	BTF: No target (7) Uppsala: No target, but clinical evaluation if cerebral glucose < 0.5 mM (MD) (11) MD consensus meeting 2014: Cerebral glucose > 0.2–0.8 mM (12)	CBF and arterial glucose management
Cerebral energy metabolism	BTF: No target (7) Uppsala: No target, but clinical evaluation if cerebral LPR > 40 (MD) (11) MD consensus meeting 2014: Cerebral LPR < 25–40 (12)	Optimize the variables above.

*BTF, Brain Trauma Foundation; CBF, Cerebral blood flow; CPP, Cerebral perfusion pressure; CPPopt, Optimal CPP; CSF, Cerebrospinal fluid; ICP, Intracranial pressure; LPR, Lactate-/pyruvate ratio; MD, Microdialysis*.

### Cerebral Perfusion Pressure—Step 2

Cerebral perfusion pressure (CPP), defined as the pressure gradient between the mean arterial blood pressure (MAP) and ICP, is another important treatment target in TBI during NIC (Tables 1, 2, Figure 1). CPP is considered a surrogate measure for global cerebral blood flow (CBF). Early studies stressed the importance of keeping CPP at high levels above 70 mmHg (18) to avoid cerebral ischemia and counteract the vasodilatory cascade. However, the Lund concept suggested that CPP values as low as 50 mmHg can usually be tolerated, whereas values above 70 mmHg may induce brain edema (8). Today, the current guidelines recommend a CPP between 60 and 70 mmHg as a balance between the risk of cerebral hypo- and hyperperfusion (7).

### Systemic Monitoring Variables

**Figure 2:**
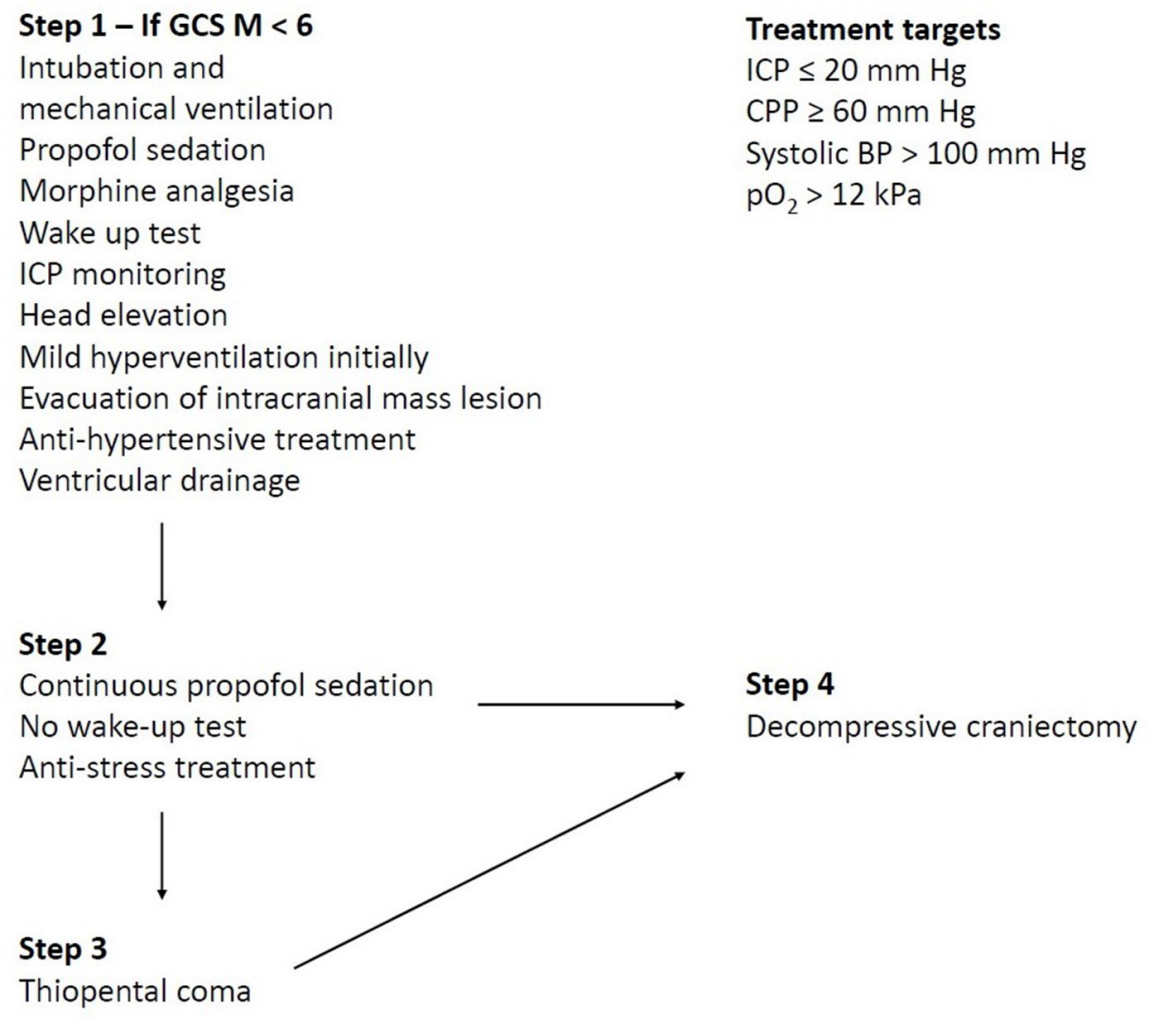
The Uppsala standardized traumatic brain injury (TBI) management protocol as an example of a local escalated intracranial pressure (ICP)-oriented management protocol with a focus on avoiding secondary insults. The figure demonstrates an example of a local ICP-oriented management protocol (2). The therapeutic intensity is gradually increased by starting with ICP-lowering treatments that carry a lower risk of complications and escalating to treatments with greater risks if the ICP target cannot otherwise be controlled. Decompressive craniectomy may be performed both on a primary indication (first surgical procedure, i.e., the fourth step is used as the first step) in the case of severe brain edema in the early course, but chiefly on a secondary indication (when all other treatments are exhausted).

In traditional NIC of TBI, ICP and CPP have been the two main treatment targets, but other variables have also been considered, e.g., to maintain sufficient arterial oxygenation (pO_2_ ≥ 12 kPa), to avoid systemic hyperthermia (*T* < 38°C), and to keep arterial glucose within normal limits (5–10 mM) (2, 19). Figure 2 demonstrates the Uppsala standardized TBI management protocol as an example of a local escalated ICP-oriented management protocol with a focus on avoiding secondary insults (2, 11, 19).

## Indicative Observations and Future Perspectives

### Multimodality Monitoring of the Complex Pathophysiology in Traumatic Brain Injury

The traditional NIC treatment targets in TBI, ICP, and CPP, are two important surrogate measures of the cerebral environment, but many other variables are also crucial steps in the pathway to achieve an optimal cerebral energy metabolic state (Figure 1). CBF delivers oxygen and glucose to the injured brain. The autoregulation of CBF by the cerebral vessels is often disturbed after TBI and focal CBF disturbances may occur despite normal ICP and CPP (20–24). In addition, systemic traumatic injuries could precipitate for arterial hypoxia with secondary brain tissue hypoxia despite normal CBF in the large cerebral vessels. Furthermore, microvascular thrombosis and diffusion limitations from brain edema may limit oxygen delivery in the microvascular circulation despite normal macrovascular CBF and arterial oxygenation (25, 26). However, even if brain tissue oxygenation and the level of energy metabolites are at adequate levels, cerebral energy failure may still occur due to mitochondrial dysfunction (26–28). In addition, mechanisms such as seizures and hyperthermia increase energy consumption and may be detrimental if the compensatory cerebral energy reserve is compromised (29–33).

Multimodality monitoring makes it possible to continuously surveil the different steps in the pathway to cerebral energy metabolism (Tables 1, 2 and Figure 1). The methods used are both global and focal, as well as continuous and intermittent. Some methods give direct information about the crucial parameters for the energy metabolism and others provide indirect measures. The complete picture is obtained by an integrated analysis of all measures. The future direction of NIC is a more detailed analysis of each of the crucial pathways for cerebral energy metabolism using multimodality monitoring in order to identify the correct cause of energy metabolic disturbances (Table 3) (34). This may in turn lead to a more timely and cause-specific treatment. The following sections will review the background and potential future applications of some monitoring techniques for the fine-tuning of next-generation NIC.

**Table 3:** Secondary brain injury conditions, multimodality monitoring patterns and potential cause-specific treatments.

**Condition**	**Multimodality monitoring pattern**	**Potential treatments**
Intracranial hypertension	High ICP, low CPP, brain tissue hypoxia, and high LPR	ICP lowering treatments
Arterial hypotension	Normal ICP, low ABP/CPP, brain tissue hypoxia, and high LPR.	Address cause for arterial hypotension, give intravenous fluids and vasopressors. Arterial hyperoxia treatment?
Autoregulatory disturbances	Normal to high ICP, low to high CPP, high PRx, brain tissue hypoxia, and high LPR.	Target CPPopt, treat high ICP, keep arterial glucose, pO_2_ and pCO_2_ within optimal intervals.
Oxygen diffusion limitation	Normal ICP, normal CPP, brain tissue hypoxia, and high LPR.	Arterial hyperoxia treatment.
Cerebral hypermetabolism	Normal ICP, normal CPP, possibly brain tissue hypoxia, low cerebral glucose and pyruvate, and high LPR.	Body temperature and seizure control. Sedation. Arterial hyperoxia treatment?
Mitochondrial dysfunction	Normal ICP, normal CPP, normal brain tissue oxygenation, normal cerebral glucose and pyruvate, and high LPR.	Arterial hyperoxia? Cyclosporin A?

*The table illustrates different multimodality monitoring patterns for different clinical conditions, including e.g., cerebral ischemia with delivery failure of cerebral energy metabolites, as well as when the cerebral energy metabolic state is compromised due to hypermetabolism or poor mitochondrial function*.

### CBF Pressure Autoregulation and CPP Management—Step 2

Global and regional CBF can be measured intermittently with different imaging techniques, and regional CBF can also be measured continuously by means of an intraparenchymal probe (Table 1). It would be advantageous to continuously monitor global CBF, but there is, at present, no feasible way. CPP is currently the best surrogate measure for global CBF, but this concept leaves the cerebrovascular reactivity out of the equation, and there is an interest to find better surrogate measures of CBF that takes both CPP and the cerebrovascular status into account.

Lassen described the cerebral autoregulation in 1959 as he demonstrated that CBF is maintained over a wide range of MAPs (35, 36). The autoregulatory capacity may become deranged following TBI, which is strongly associated with poor outcome (14, 21, 37). Several methods to monitor the cerebral autoregulation in the NIC have been introduced (Table 1), such as the mean flow index (Mx) and the pressure reactivity index (PRx). PRx has received the greatest interest and is continuously measured as the correlation coefficient between MAP and ICP over 5 min (37). Negative index values indicate intact pressure autoregulation, such as when an increase in MAP leads to cerebral vasoconstriction to maintain normal CBF with a corresponding reduction in CBV and ICP. Positive index values indicate disturbed pressure autoregulation, such as when an increase in MAP leads to passive cerebral vasodilation with corresponding increases in CBF, CBV, and ICP (37–39). The clinical utility of PRx is still under development. However, as low PRx values are associated with better clinical outcome, autoregulatory-oriented management that aims at improving PRx has been suggested, as outlined below.

**Figure 3:**
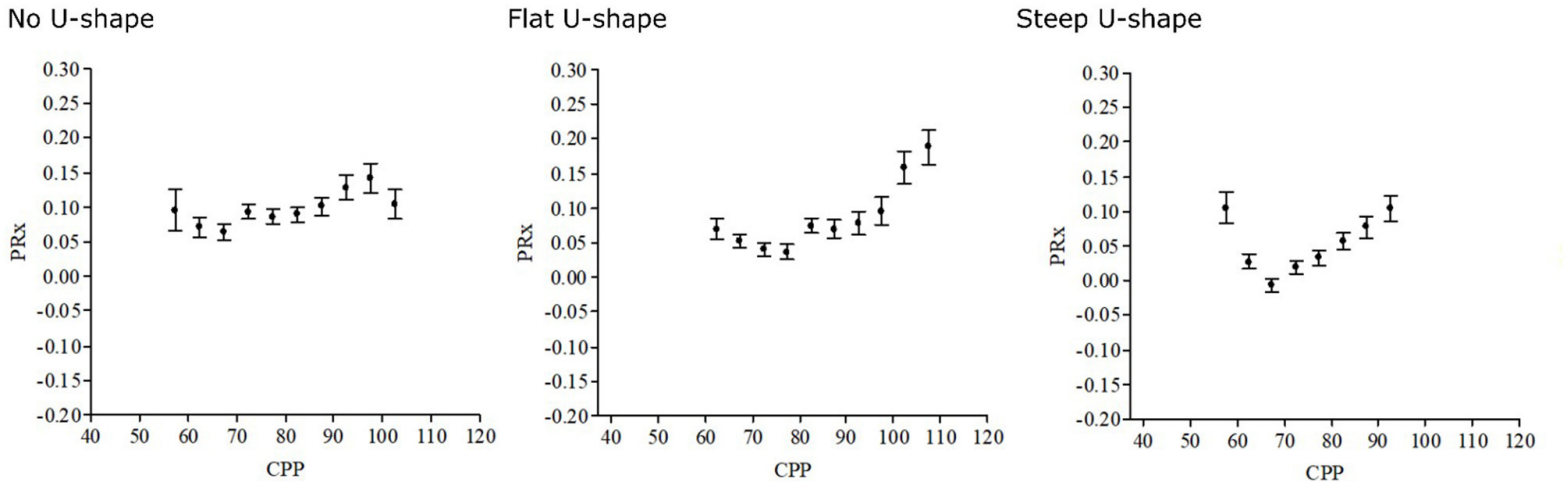
Optimal cerebral perfusion pressure (CPPopt)—differences in curve shape. The CPPopt curves may vary over time and between patients, as illustrated in these three different curves. There are questions on how the curve shape and the absolute pressure reactivity index (PRx) are relevant in CPPopt management. Patients with a steeper CPPopt curve may, in theory, be more vulnerable to changes in CPP.

Autoregulatory management aims at giving the patient treatments that improve the autoregulatory capacity. Particularly, the association between pressure autoregulation and CPP has received interest. In a first attempt to consider autoregulation in CPP management, patients were classified as either pressure active or pressure passive, and the absolute autoregulatory status was suggested to determine whether the patient would benefit from high or low CPP (40). It was then demonstrated in an observational study including two centers with different CPP philosophy (Uppsala and Edinburgh) that patients with pressure-passive cerebral vessels (high PRx) had better outcomes if treated with ICP-oriented therapy with relatively lower CPP targets, whereas pressure-active patients (low PRx) benefitted from CPP-oriented treatment with relatively higher CPP targets (41). In the next attempt, it was found that PRx varies with CPP in a U-shaped way and that the CPP with the concurrently lowest PRx could be targeted where autoregulation works best (22), as demonstrated in Figure 3. Several studies have supported that deviation of the absolute CPP above and below the optimal (CPPopt) is associated with poor outcomes (24, 42–45). Furthermore, brain tissue oxygenation reaches a plateau when CPP approaches CPPopt, indicating optimal CBF (46). These findings support that CPPopt may be a better CBF surrogate that takes into account both the absolute CPP and the cerebrovascular status for the individual patient.

However, despite these promising findings, several concerns have been raised regarding CPPopt that need to be addressed in future trials. The U-shaped association between CPP and PRx is absent during ~40% of the monitoring time (47), which limits the feasibility of CPPopt as a target (48). Secondly, the appearance of the U-shaped curve may vary from flat to steep and the absolute PRx at CPPopt may vary from negative to positive values (Figure 3). The necessity to keeping CPP close to CPPopt probably depends on the curve shape and the absolute PRx (42). For example, if CPPopt is high, the absolute PRx is negative, and the U-shaped curve is relatively flat, the Lassen plateau phase of autoregulation should be acceptably wide and a much lower absolute CPP below CPPopt could then be allowed since higher targets are otherwise associated with complications such as acute respiratory distress syndrome (49). On the contrary, it could be more critical to keep CPP close to CPPopt if the curve is steep and PRx is high. Thirdly, although patients that spontaneously had CPP values close to CPPopt had better clinical outcome in retrospective studies (43), CPPopt as an active treatment target has so far not been sufficiently investigated prospectively (50). Particularly, CPPopt is often above 70 mmHg, i.e., above the upper fixed threshold according to the BTF guidelines (7), but using vasopressors to achieve such high targets could carry adverse effects. The safety and feasibility of CPPopt as a treatment target is investigated in a multicenter randomized controlled trial (COGiTATE) (9). The results of this trial will determine the future directions of CPP research and management in TBI care.

### Autoregulatory Management and Systemic Physiology—Step 2

In addition to CPP, several other variables that control the cerebrovascular resistance are associated with pressure autoregulation and could potentially be targeted in an integrated autoregulatory protocol. Arterial hyperglycemia may disturb the endothelial and myogenic function of the cerebral vessels (51, 52). In line with these findings, we and others have demonstrated that arterial hyperglycemia is independently associated with a higher PRx (53, 54), indicating that a tighter glycemic control at least below 10 mM could be beneficial from an autoregulatory point of view. Furthermore, lactate is a cerebral vasodilator and higher systemic levels may increase CBF (55). However, we have found that a higher arterial lactate is independently associated with a higher PRx (33), and it is possible that the corresponding increase in CBF (55) represents dysregulated hyperemia. This calls for caution in clinical TBI trials on lactate-based fluids as a way of increasing the delivery of an alternative energy fuel to the brain due to the possible negative effect of lactate on pressure autoregulation. Hyperthermia could induce cerebral vasodilation, and some findings support an association between a higher body temperature and a higher PRx (56), whereas other studies have found no such association (33). Arterial hypoxia also leads to cerebral vasodilation in order to increase CBF to maintain normal brain tissue oxygenation (57), whereas higher oxygen levels may increase the cerebrovascular tone. We have found that higher arterial oxygen levels above 12 kPa are associated with lower PRx, i.e., better pressure autoregulation (58). Similarly, increased cerebrovascular tone by hyperventilation is associated with better pressure autoregulation (56, 59, 60).

Hence, several systemic variables, in addition to CPP, could potentially be targeted in an integrated autoregulatory protocol including, e.g., temperature management, glycemic control, and respiratory targets to optimize pO_2_ and pCO_2_.

### Arterial and Brain Oxygenation—Steps 3 and 4

Ischemic and hypoxic secondary brain injuries are common after TBI (61), and treatment aims at maintaining adequate CBF and arterial oxygen content for aerobic energy metabolism. Arterial oxygen content is monitored with repeated arterial blood gas analyses of hemoglobin (Hgb) and pO_2_ and continuously with pulse oximetry (SpO_2_). The arterial oxygen content is, to a great extent, dependent on the level of oxygen-binding Hgb, whereas dissolved oxygen only constitutes a fraction of the total arterial oxygen content under normal conditions (62). There is great controversy regarding the definition of clinically relevant anemia for cerebral oxygen delivery, but an Hgb below 9 g/dl may discriminate when the risk of cerebral hypoxia is significantly increased (63). Although red blood cell transfusion (RBCT) could improve cerebral oxygen delivery (64), adverse effects such as a worsening in pressure autoregulation (65) and an increased risk of thromboembolic events have been described (66). Clinical studies on liberal *vs*. restricted RBCT based on Hgb thresholds (e.g., 10 vs. 7 g/dl) indicate a neutral to negative effect on clinical outcomes with the liberal approach (64, 66–69). The observations that both anemia and RBCT are independently associated with poor outcomes make the clinical management difficult, but it is possible that the indication for RBCT could be guided by means of multimodal monitoring. Despite, or because, the controversy in this matter, there is currently no BTF guidelines on RBCT in severe TBI (7).

There is also limited knowledge and lack of guidelines regarding specific arterial oxygen thresholds (7), but pO_2_ levels above 12 kPa and SaO_2_ above 95% may be targeted (2). Additionally, brain tissue oxygenation can be monitored globally with a jugular vein bulb (SjvO_2_) and focally with an intraparenchymal probe (BtO_2_) (Table 1). BTF currently recommends SjvO_2_ monitoring and avoiding ischemic SjvO_2_ levels below 50% (7). There is no BTF recommendation on BtO_2_ monitoring (7), but focal brain tissue hypoxia below 20 mmHg has been associated with poor clinical outcomes (70).

Although there is only a small fraction of free and dissolved pO_2_ in the blood (62), an increase in pO_2_ to hyperoxic levels could still be beneficial in TBI. Increasing the fraction of the inspired oxygen (FiO_2_), i.e., normobaric hyperoxia (NBO), has been proven to increase arterial and brain tissue oxygenation (71). NBO may then compensate for ischemic hypoxia, overcome diffusion barriers, and improve mitochondrial function (62). Hyperoxia reduces cerebral glycolytic enzymes and thereby decreases pyruvate and lactate (57, 71, 72), but there are questions whether cerebral oxidative energy metabolism improves. A PET study found that hyperoxia improves energy metabolism in ischemic brain regions (73), and microdialysis (MD) studies support true energy metabolic improvements in the case of poor substrate supply (58) or anaerobic energy metabolism (74). On the other end, some studies suggest that hyperoxia induces detrimental reactive oxygen species that negatively affect cerebral tissue survival, although this may only occur to a small extent in a limited number of patients (75). Brain tissue oxygen-guided treatment protocols have been introduced, in which low brain tissue oxygenation may be treated with an increase in FiO_2_. However, there is still clinical equipoise of this approach due to small positive to neutral effects on clinical outcomes in prospective trials (76–78).

### Delivery of Cerebral Energy Metabolites, Cerebral Energy Metabolism, Neurochemical Monitoring, and Treatments—Steps 3, 4, and 5

Cerebral nutrients, such as glucose and lactate, are delivered by the CBF. The arterial levels may be monitored with repeated arterial blood gases to ensure sufficient arterial content. The cerebral energy metabolites, glucose, pyruvate, and lactate, as well as the rate of oxidative energy metabolism (lactate/pyruvate ratio, LPR) may be evaluated with hourly measures by means of cerebral MD, i.e., a double-lumen, semi-permeable catheter that is perfused with artificial CSF (79, 80). The catheter can either be placed in macroscopically normal-appearing brain tissue to estimate global cerebral energy metabolism or in peri-contusional areas to estimate focal cerebral energy metabolism in “tissue-at-risk” (81). Recently, the new MD system (Loke) has been introduced, which measures cerebral glucose, pyruvate, and lactate minute by minute. This may reveal the complex explanatory variables for cerebral energy metabolism in higher resolution. The cerebral energy metabolism may also be evaluated with radiological snapshots using PET (Table 1).

Glucose is the main cerebral energy fuel. The arterial glucose level and CBF determine cerebral glucose delivery, but the cerebral glucose level also depends on the cerebral energy metabolic rate. Arterial and cerebral glucose are normally correlated, but this association may be disturbed after TBI (33, 54, 82–84) as the CBF and cerebral energy metabolic rate could have greater influence on cerebral glucose levels. The immediate effects of TBI include a sympathetic stress response that, among other things, gives rise to a surge in arterial glucose (85). This is beneficial since it is necessary to avoid arterial hypoglycemia and neuroglycopenia for the vulnerable brain, but arterial hyperglycemia is also associated with worse outcomes after TBI (86–88). Although the latter association could reflect more severe underlying traumatic injuries, hyperglycemia could, *per se*, induce secondary brain injury by causing disturbances in cerebral autoregulation and mitochondrial function (54, 89). Since both too low and too high arterial glucose could exert a negative effect on the brain, tight glycemic control with intensive insulin therapy (IIT) management has been suggested in TBI care. IIT showed promising results in general ICU patients at an early stage (10, 90, 91), but recent studies show that IIT causes an increased burden of arterial hypoglycemia that outweighs the benefits of avoiding hyperglycemia, resulting in a neutral to negative net effect on outcomes in various ICU populations as well as in TBI (92–97). Specifically, TBI patients treated with IIT developed more severe energy metabolic disturbances with reductions in cerebral glucose and oxidative energy metabolism than those treated with conventional glycemic control (95, 98). Hence, a narrow arterial glucose interval is appealing in theory, but is currently not feasible due to the risk of overtreatment. BTF does not have any recommendation on optimal glucose levels or management in TBI (7). Due to the lack of clear evidence of benefits for IIT in the NIC setting, a looser glycemic control is mostly applied, e.g., between 5 and 10 mM (99). Cerebral MD glucose below 0.2–0.8 mM is considered dangerously low, and such thresholds could aid in guiding treatment for when the termination of insulin treatment or infusion of glucose is warranted (12).

The understanding of arterial lactate as an additional cerebral energy substrate (100) has increased in the last years. However, the arterial contribution to cerebral lactate is only 10% when the arterial lactate concentration is 1 mM, but increases to 60% at 5 mM (101). An increased consumption of lactate as an energy fuel may, in turn, spare cerebral glucose (55, 102, 103). Higher lactate decreases the cerebrovascular tone, induces vasodilation, and increases CBF (55, 104), but possibly at the expense of a worsened pressure autoregulation (33). Exogenous, hypertonic lactate may exert anti-edematous effects and alleviate intracranial hypertension (102). Animal TBI studies support a reduction in lesion volume after exogenous lactate infusion (105). Clinical trials also indicate that exogenous lactate improves outcomes (106), although a higher endogenous arterial lactate is independently associated with worse outcomes after TBI (33). Future studies are needed to determine the role of intravenous lactate supplement in TBI care. Nevertheless, high endogenous levels of cerebral lactate above 4 mM indicate anaerobic energy metabolism and are associated with unfavorable clinical outcomes (12, 107).

Furthermore, high cerebral MD LPRs above 25–40 indicate disturbed cerebral energy metabolism (12, 107). The etiology for LPR elevations may differ. A high LPR with concurrently low cerebral glucose and pyruvate has been suggested to reflect cerebral ischemia/substrate delivery failure, whereas a high LPR with concurrently normal/high cerebral glucose and pyruvate has been suggested to reflect mitochondrial dysfunction (26). Substrate delivery could, in theory, be improved by a higher CPP, improved pressure autoregulation, and higher arterial substrate content (Table 3). The importance of mitochondrial dysfunction has gained increased interest and understanding in recent years. There is currently no treatment for this condition, but cyclosporine A, which is an immunosuppressant, improves the mitochondrial function in animal TBI models and has demonstrated some promise in preliminary human trials, but larger studies are needed to evaluate its efficacy (108, 109).

MD may also aid in monitoring more complex dynamic pathophysiological processes in the brain following TBI. Neuroinflammation after TBI may exert both beneficial and negative effects on the brain, depending on the time window after injury and the specific mechanisms (110). MD can be used to evaluate neuroinflammatory biomarkers bedside in order to better understand the neuroinflammatory mechanisms, their relation to the dynamic clinical course, secondary insults, and clinical outcomes in TBI (111, 112). This may aid in the development of optimal neuroprotective agents, as outlined below, and the cerebral MD biomarkers could potentially be used to improve the indication for such treatments.

### Neuroprotective Agents

In addition to the secondary physiological insults, there is a cascade of injury mechanisms on the cellular and molecular levels after the primary brain injury, e.g., toxic release of excitatory neurotransmitters, apoptotic pathways, neuroinflammation, and mitochondrial dysfunction (113). Many neuroprotective agents have been developed to counteract these pathomechanisms, but there has so far been limited success in translation from animal to clinical studies (113). For example, *N*-methyl-d-aspartate (NMDA) receptor antagonists and calcium channel antagonists both reduce excitotoxicity in animal TBI models, but their efficacy in human TBI trials has been limited (114, 115). Human TBI trials on immunomodulating agents have also been disappointing. Progesterone had a neutral effect (116), whereas corticosteroids even worsened clinical outcomes (117). As outlined above, cyclosporine A has demonstrated some promise in preliminary human trials, but larger studies are needed to evaluate its efficacy (108, 109).

One reason for the so far disappointing results of neuroprotective drugs in humans may be that the therapeutic time window is limited, and it may be difficult to administer the treatments within the right time in the clinical setting. Furthermore, TBI in humans is very heterogeneous, and the subtype may be of importance for the therapeutic effect. Animal trials are often based on relatively homogenous TBI models, while the clinical trials often have included all types of TBI patients. Many pharmacological trials have also been done in the dawn of NIC, when secondary insults were not yet as efficiently surveyed and managed as today, and the relative importance of these agents might have been less significant in that setting. In conclusion, despite the limited effect of the neuroprotective agents shown in human TBI trials, it is possible that neuroprotective agents with a more optimal treatment timing, better understanding of the injury processes, and an improved patient selection in a high-quality NIC setting could yield improved outcomes. Future studies that take these aspects into account are needed.

### Multimodality Monitoring and Precision Medicine

**Figure 4:**
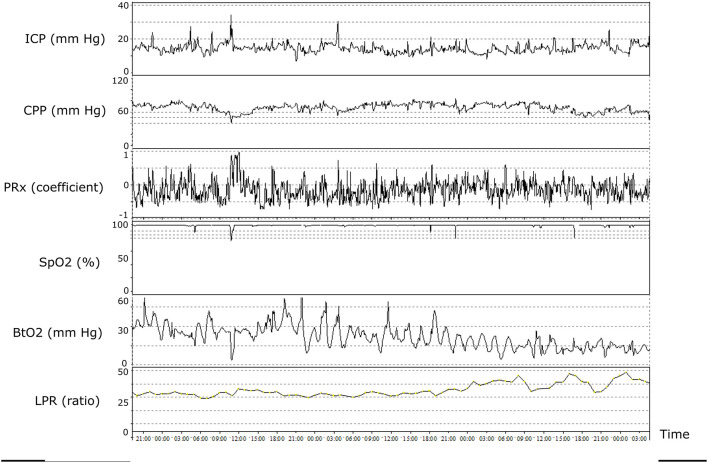
Illustrative patient case with multimodal monitoring. The figure demonstrates the temporal evolution of cerebral physiology over ~2 days. Although intracranial pressure (ICP), cerebral perfusion pressure (CPP), pressure reactivity index (PRx), and oxygen saturation (SpO_2_) were within adequate intervals, the patient developed brain tissue hypoxia and cerebral energy failure (high lactate/pyruvate ratio). The pathophysiology may include microvascular thrombosis and/or increased diffusion limitations from cerebral edema.

The multimodality monitoring pattern may reveal the underlying etiology of cerebral energy failure following TBI so that cause-specific treatments may be initiated (Table 3). For example, in cases of intracranial hypertension, low CPP, brain tissue hypoxia, and cerebral energy failure, treatments should aim at lowering ICP to improve the cerebral environment. In other cases with normal ICP, CPP within optimal targets, and normal arterial oxygenation, but poor brain tissue oxygenation and concurrent cerebral energy metabolic failure (Figure 4), arterial hyperoxia could be used to possibly overcome cerebral diffusion barriers. In cases where all variables such as ICP, CPP, and brain tissue oxygenation are within the targeted intervals, except for the energy metabolic state (LPR), mitochondrial dysfunction is a plausible explanation. There is currently no treatment for this condition, but there are ongoing trials for, e.g., neuroprotective agents that may relieve mitochondrial dysfunction.

### Cerebral Monitoring in a Resource-Limited Setting

High-resolution multimodal monitoring offers the best method to fully evaluate the cerebral environment after TBI, but such tools may be limited to a few research-minded academic centers. There is, hence, also an interest in more feasible monitoring tools. PRx and CPPopt both require high-resolution data and advanced software. However, low-frequency autoregulation index (LAx) and the corresponding LAx-derived CPPopt are based on minute-by-minute data, require less advance software, and may be a fair substitute for centers without access to PRx and CPPopt (118). Transcranial Doppler is also a feasible noninvasive and accessible method to assess cerebral blood flow velocity, particularly in the absence of CPP and advanced radiological imaging, and could be used to guide blood pressure management.

## Concluding Remarks

Traditional NIC has focused on reducing secondary brain injury by treating elevated ICP and maintaining the CPP sufficiently high to avoid cerebral ischemia. However, recent findings support that the TBI pathophysiology is much more complex and cerebral energy failure frequently occurs in the absence of intracranial hypertension and low CPP. Particularly, the roles of pathomechanisms such as disturbances in pressure autoregulation, microvascular thrombosis, oxygen diffusion limitations, and mitochondrial dysfunction have gained increased understanding. By multimodal monitoring, cerebral energy metabolic failure may be detected earlier and its etiology could be better diagnosed. This may, in turn, lead to precision medicine with more cause-specific treatments to avoid secondary brain injury for these patients. Future studies are needed to elaborate on the strengths and limitations of certain monitoring tools and their role in guiding cause-specific treatments in NIC of severe TBI.

## Author Contributions

TS: conceptualization, writing–original draft. AL: conceptualization, writing–review and editing. PE: conceptualization, supervision, writing–review and editing. All authors contributed to the article and approved the submitted version.

## Conflict of Interest

The authors declare that the research was conducted in the absence of any commercial or financial relationships that could be construed as a potential conflict of interest.
